# Quantitative MR thermometry based on phase-drift correction PRF shift method at 0.35 T

**DOI:** 10.1186/s12938-018-0472-x

**Published:** 2018-04-10

**Authors:** Yuping Chen, Mengke Ge, Rizwan Ali, Hejun Jiang, Xiaoyan Huang, Bensheng Qiu

**Affiliations:** Centers for Biomedical Engineering, University of Science and Technology of China, Hefei, 230026 Anhui China

**Keywords:** Quantitative MR thermometry, Proton resonance frequency shift, Phase-drift correction, Microwave ablation, Temperature mapping

## Abstract

**Background:**

Noninvasive magnetic resonance thermometry (MRT) at low-field using proton resonance frequency shift (PRFS) is a promising technique for monitoring ablation temperature, since low-field MR scanners with open-configuration are more suitable for interventional procedures than closed systems. In this study, phase-drift correction PRFS with first-order polynomial fitting method was proposed to investigate the feasibility and accuracy of quantitative MR thermography during hyperthermia procedures in a 0.35 T open MR scanner.

**Methods:**

Unheated phantom and ex vivo porcine liver experiments were performed to evaluate the optimal polynomial order for phase-drift correction PRFS. The temperature estimation approach was tested in brain temperature experiments of three healthy volunteers at room temperature, and in ex vivo porcine liver microwave ablation experiments. The output power of the microwave generator was set at 40 W for 330 s. In the unheated experiments, the temperature root mean square error (RMSE) in the inner region of interest was calculated to assess the best-fitting order for polynomial fit. For ablation experiments, relative temperature difference profile measured by the phase-drift correction PRFS was compared with the temperature changes recorded by fiber optic temperature probe around the microwave ablation antenna within the target thermal region.

**Results:**

The phase-drift correction PRFS using first-order polynomial fitting could achieve the smallest temperature RMSE in unheated phantom, ex vivo porcine liver and in vivo human brain experiments. In the ex vivo porcine liver microwave ablation procedure, the temperature error between MRT and fiber optic probe of all but six temperature points were less than 2 °C. Overall, the RMSE of all temperature points was 1.49 °C.

**Conclusions:**

Both in vivo and ex vivo experiments showed that MR thermometry based on the phase-drift correction PRFS with first-order polynomial fitting could be applied to monitor temperature changes during microwave ablation in a low-field open-configuration whole-body MR scanner.

## Background

Magnetic resonance thermometry (MRT) is a non-invasive technique for monitoring tissue temperature during tumor thermotherapeutic procedures [1, 2]. The mechanism of MR temperature mapping is based on temperature sensitive magnetic resonance parameters, such as T1 and T2 relaxation times, water diffusion coefficient, PRFS, proton density, magnetization transfer and temperature sensitive contrast agents [3–9]. Compared with other methods, PRFS has acceptable linearity and it is near independent with regards to tissue type except adipose tissue, which makes it a good choice for monitoring temperature changes [8, 10].

Minimally invasive interventional procedures are an option to relieve pain and minimize the risk of disability. Laser induced interstitial thermotherapy (LITT) [1, 2, 11–13], high intensity focused ultrasound (HIFU) [13–15], radiofrequency (RF) [13, 16–18] and microwave (MW) [13, 19, 20] are conventional thermal ablation methods for MRT. Minimally and/or non-invasive thermal therapies ensure localized damage to pathological tissues, while at the same time having good healthy tissue-sparing capabilities. MRT is widely used to guide and monitor thermal therapy procedures because it is able to provide fast temperature mapping within the target ablation tissue with acceptable spatial resolution.

In 1966, the temperature sensitivity of the PRF was observed by Hindman for the first time [21]. Subsequently, Ishihara et al. and De Poorter et al. applied PRF shift method to monitor MR temperature [3–5]. As the temperature increases, the local magnetic filed and the proton resonance frequency become lower due to the stretching, bending and breaking of the hydrogen bond in water molecules [21]. PRFS is able to obtain the relative temperature difference (ΔT) on the basis of the phase changes (ΔΦ) of gradient echo (GRE) or spoiled-gradient echo (SPGR) sequences [3–5]. When temperature ranges from − 15 °C to 100 °C, the linear relationship between ΔT and ΔΦ can be expressed as the following formula:

1 $$\Delta {\text{T}} = \frac{\Delta \varPhi }{{\varUpsilon \alpha B_{0} TE}}$$

where ϒ = 42.58 MHz/T is the gyromagnetic ratio of H^1^, α = − 0.01 ppm/°C is the PRF thermal coefficient for aqueous tissue [21, 22], B_0_ is the main magnetic field strength, TE is the echo time of pulse sequence, ΔΦ is the difference between reference phase images acquired before heating at a known temperature and images acquired during heating cycle at different temperatures.

The phase difference was constructed by the complex calculation based on Eq. (2) rather than a simply subtraction, which could effectively avoid problematic phase wrapping during the heating cycle [23]

2 $$\Delta \varPhi = {\text{atan}}\left( {\frac{{Re\left( {I_{ref} } \right)*Im\left( {I_{H} } \right) - Im\left( {I_{ref} } \right)*Re\left( {I_{H} } \right)}}{{Re\left( {I_{ref} } \right)*Re\left( {I_{H} } \right) + Im\left( {I_{ref} } \right)*Im\left( {I_{H} } \right)}}} \right)$$

where Re and Im are the real and imaginary components of the heated (I_H_) and reference (I_ref_) images.

It is well studied that one of the biggest drawbacks of MRT based on PRFS is external magnetic field drift during long-term ablation therapy [5, 7]. To eliminate tissue motion and frequency drift, a referenceless PRF method proposed by Rieke et al. demonstrated that high-order (second-to sixth-order) polynomials could better estimate the background phase outside the heated region [10]. However, the previous method gained larger temperature errors when it was applied to monitor tissue temperature changes in low-field MRI systems. To overcome the phase difference arising from external field drift, phase-drift correction PRFS thermometry with first-order polynomial fitting was proposed to monitor temperature changes at low-field scanners. It is generally accepted that as the ablation time increases, the external magnetic field drift will increase. The MW heating duration in Sherar et al. 70, 100 and 120 s were for three rabbits [24]. To more accurately verify the performance of the phase-drift correction PRFS thermometry method, the time to ablate an in vitro porcine liver in this study was 330 s.

In the past studies, the vast majority of interventional MRT for cancer tissue ablation experiments were implemented at high-field strength, such as 1.5 and 3.0 T [1, 13, 18, 20, 25–30]. Sporadic literature had reported applicability in MR temperature measurement at low-field strength [31–35]. High field MR devices (1–3 T) are usually closed bore magnets due to the requirements of robust shielding and gradient structure to maintain field homogeneity. In contrast, low-field MR scanners (0.2–1.0 T) are more suitable for interventional hyperthermia procedures than closed systems, because they are open system with access to the patients, as well as lower purchase price and operational costs [34, 36–39]. It is of practical meaning for low-field MRT to measure the ablation temperature and area. In this study, phantom, in vivo human brain and ex vivo porcine liver MW ablations experiments demonstrated the feasibility and accurate of phase-drift correction PRFS thermometry in a 0.35 T open MR scanner.

## Methods

### Phase-drift correction PRFS thermometry

In the phase-drift correction PRFS thermometry model, the region of interest (ROI) is selected on the actual thermal target, and its size can be changed arbitrarily. Outer and inner ROI regions represent unheated and heated areas, respectively. The phase difference of the outer ROI can be able to fit the phase drifts arising from extraneous sources apart from the variation of temperature in the inner ROI (ΔΦ_f_) by a first-order polynomial, which can be written as

3 $$\Delta \varPhi_{f} \approx a_{0} + a_{1} x + a_{2} y$$

When the observational errors are uncorrelated and the weight coefficient matrix, W, is diagonal, the smooth function can be rewritten as Eq. (4).

4 $$\left( {X^{T} WX} \right)A = X^{T} W\Delta \varPhi_{f}$$

where X denotes spatial coordinates, A is the polynomial coefficients that is determined via a weighted least squares fitting. According to the phase-drift correction algorithm, conventional PRFS can be noted as:

5 $$\Delta {\text{T}} = \frac{{\Delta \varPhi - \Delta \varPhi_{f} }}{{\varUpsilon \alpha B_{0} TE}}$$

Equation (5) indicates that temperature sensitivity (ΔΦ/ΔT) is related to B_0_ and TE. At an echo time of 30 ms for a 0.35 T MR scanner, the temperature sensitivity is 1.61°/°C, which is almost 4.3 times smaller than that at 1.5 T under the same TE.

### Materials and experimental settings

All imaging experiments were performed on a 0.35 T (PICA, Time Medical Systems, Hong Kong, China) open-configuration whole-body MR scanner with head coil. A 2.45 GHz microwave ablation instrument (METI-IVD, Fuzhong Medical, Nanjing, China) was the heating device. For ex vivo porcine liver ablation experiments, a 15 cm long and 2.5 mm in diameter MR-compatible ablation antenna based on a 50-Ω UT-085 semirigid coaxial cable was used. All tissues were kept stationary for two hours before scanning to equalize with the room temperature. The study was approved by the ethics committee of our institution.

### Unheated phantom experiments

For all unheated experiments, it was assumed that the temperature of the object did not change in the course of experiments. This assumption was reasonable because the subject was not affected by the external temperature.

**Fig. 1:**
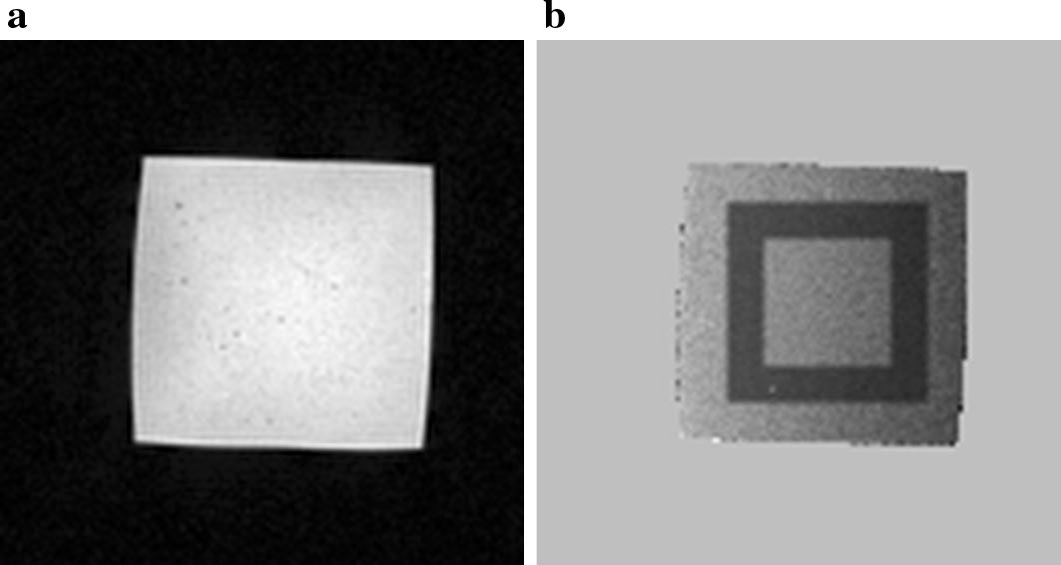
Gel phantom and ROIs selection. **a** Magnitude image of an unheated gel phantom. **b** Black region on phase difference map represents outer ROI and inner ROI is contained in outer ROI

A cubic (12 × 12 × 8 cm^3^) gel phantom which consisted of 2% agar, 4% gelatin, 0.5% NaCl and 0.05% CuSO_4_ per 1 L pure water was used in this experiment. Images were realized by GRE sequence in the coronal plane with the following scanning parameters: repetition time (TR) = 50 ms, echo time (TE) = 30 ms, flip angle (FA) = 30°, Matrix size = 128 × 128, field of view (FOV) = 220 mm, number of slices = 3, slice thickness (ST) = 8 mm, slice gap (SG) = 0 mm, acquisition time = 6.4 s/slice. Five images were acquired sequentially without heating to verify the feasibility of MRT. The selected inner ROI contains 30 × 30 pixels, approximately 5.2 × 5.2 cm^2^. As seen in Fig. 1b, the width of outer ROI was 10 pixels (about 1.7 cm) on every side outside the inner ROI.

### Unheated ex vivo porcine liver experiments

**Fig. 2:**
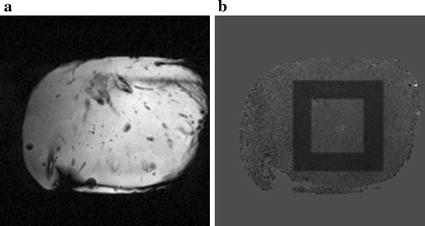
Ex vivo porcine liver and ROIs selection. **a** Magnitude image of the unheated porcine liver. **b** Inner and outer ROIs on phase difference image

Unheated experiments were performed on an ex vivo porcine liver at 0.35 T with TR/TE = 50/30 ms, FA = 30°, Matrix size = 160 × 160, FOV = 256 mm, number of slice = 1, ST/SG = 8/0 mm, acquisition time = 8 s/slice. Only one slice of data was scanned five times due to the irregular shape of porcine liver. The selected inner ROI contains 40 × 40 pixels, approximately 6.4 × 6.4 cm^2^. Magnitude image from ex vivo porcine liver was depicted in Fig. 2a. As seen in Fig. 2b, the width of outer ROI was on every side outside the inner ROI. In this part, three different size of ROIs were set to determine the impact of ROIs selection on temperature errors. The inner and outer ROIs contain 30 and 15 pixels for ROI selection (RS) 1, 40 and 15 pixels for RS 2, and 30 and 10 pixels for RS 3, respectively.

### Ex vivo porcine liver ablation experiments

A 2.45 GHz microwave generator was positioned outside the scanner room and the output power was set at 40 W for 330 s. Independent temperature measurements were obtained by fiber optic temperature probe (PalmSense, Photon Control, Inc, Burnaby, B.C., CA). MR-compatible ablation antenna was inserted into the ex vivo porcine liver. The fiber optic temperature probe, surrounded by the catheter, was oriented parallel to the scanning plane. Images were acquired during microwave ablation procedure by GRE sequence in the coronal plane using the following scanning parameters: TR/TE = 50/30 ms, FA = 30°, Matrix size = 128 × 128, FOV = 200 mm, number of slices = 1, ST/SG = 8/0 mm, acquisition time = 6.4 s/slice. The width of the selected inner and outer ROI were 25 (about 3.9 cm) and 10 pixels (about 1.6 cm), respectively. The ex vivo porcine liver was kept stationary to avoid the effects of movement. In this part, relative temperature changes calculated by the phase-drift correction PRFS method were compared to the temperature values measured by fiber optic temperature probe at the same pixels in order to reveal the accuracy of the algorithm of MRT.

### In vivo human brain experiments

**Fig. 3:**
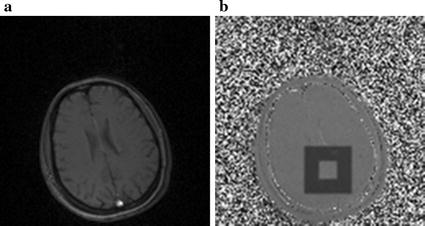
Human brain and ROIs selection. **a** Magnitude image. **b** Inner and outer ROIs on phase difference image

The feasibility and repeatability of the phase-drift correction PRFS thermometry was assessed by brain temperature experiments of healthy volunteers at room temperature. Three healthy volunteers were involved in this study and informed consent was written before accepting examinations. Images were scanned by GRE sequence in the transverse plane using the following scanning parameters: TR/TE = 50/30 ms, FA = 30°, Matrix size = 128 × 128, FOV = 256 mm, number of slices = 3, ST/SG = 8/0 mm, acquisition time = 6.4 s/slice. In order to compare with the results obtained by Zou et al. at 3.0 T MR scanner [40], the width of the selected inner and outer ROI (see as Fig. 3b) were both 10 pixels (2 cm), which was the same as the above published literature. The measurements were repeated four times to ensure the accuracy and security of the method for clinical interventional therapy. Student’s t test was used in statistical analysis to compare the root mean square error (RMSE) of temperature error between three volunteer studies.

### Data analysis

All images were processed and analyzed off-line by MatLab (R2015a, The MathWorks, Inc, Natick, MA, USA) and ran on a computer with 8 GB of RAM and four core CPU operating at 3.3 GHz. Phase difference mappings were calculated by subtracting the reference phase image from the phase images acquired during the heating process. In the unheated experiments, the temperature RMSEs within the inner ROIs, calculated by conventional and first-to sixth-order polynomial fitting, were compared to choose the best order for phase-drift correction PRFS thermometry. The order of the polynomial to obtain the smallest temperature error was chosen as optimal. For microwave ablation experiments, temperature profile calculated by the phase-drift correction PRFS was compared with the temperature values resulted from the averaging over a nine pixels neighborhood surrounding the tip of the fiber optic temperature probe in the target thermal region. In addition, temperature difference values for unheated experiments could be entailed by the phase difference mappings using theoretical PRF thermal coefficient of − 0.01 ppm/°C [32].

## Results

### Unheated phantom experimental results

**Fig. 4:**
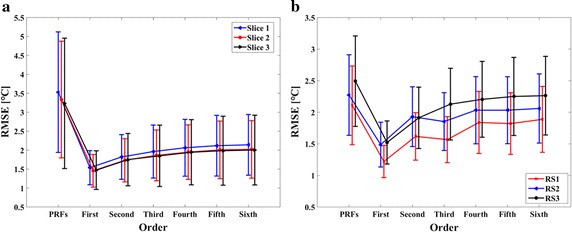
The temperature RMSE (Mean ± SD) in the inner ROI. **a** Unheated phantom. **b** Unheated ex vivo porcine liver. RS1, RS2 and RS3 represented that the inner ROI contained 30, 40 and 30 pixels and the outer ROI contained 15, 15 and 10 pixels, respectively

The temperature change in the inner ROI should be 0 °C in unheated experiments and any MR measured temperature rise was considered as measurement error. The temperature RMSE in the inner ROI was computed to determine the optimal weighted least square (WLS) polynomial fitting order for eliminating the phase difference arising from extraneous sources. The mean and standard deviation (SD) value of the temperature RMSEs computed by conventional PRFS were all bigger than those calculated by the phase-drift correction PRFS. The improvement of the phase-drift correction PRFS using first-order polynomial fitting method was particularly obvious in each slice, whose temperature RMSEs were 1.54 ± 0.45, 1.46 ± 0.43 and 1.47 ± 0.51 °C, respectively (Fig. 4a).

### Unheated ex vivo porcine liver experimental results

The mean and SD of the temperature RMSE in the inner ROI for three different size of ROIs, which were calculated by conventional PRFS and phase-drift correction PRFS with first-to sixth-order polynomial fitting (Fig. 4b). In the case of three different ROIs selection, the temperature RMSE calculated by phase-drift correction PRFS with first-order polynomial fitting had an obvious advantages over high-order polynomial fitting and the original PRF methods, and its mean RMSEs were 1.22, 1.49 and 1.52 °C for RS1, RS2 and RS3, respectively. Therefore, temperature differences of ex vivo porcine liver during the heating process would be calculated by the phase-drift correction PRFS with first-order polynomial fitting.

### Ex vivo porcine liver ablation experimental results

**Fig. 5:**
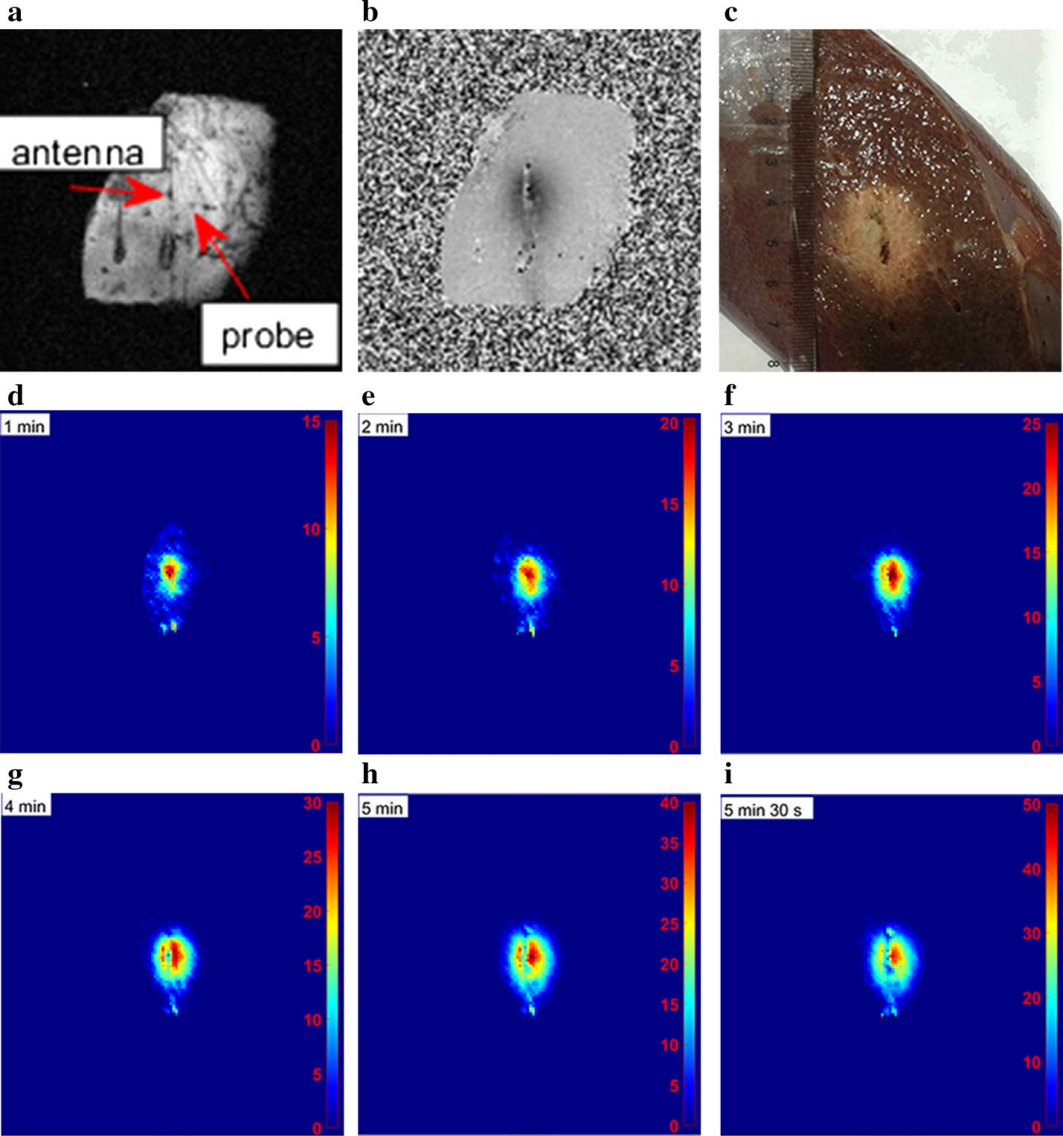
**a** Magnitude image of ex vivo porcine liver acquired before ablating (red arrows indicated microwave ablation antenna and probe, respectively). **b** Phase difference image. **c** Ex vivo porcine liver after microwave ablation. **d**–**i** Relative temperature maps for ex vivo porcine liver at six time points during microwave ablation procedure

In order to obtain a stable starting temperature value, ex vivo porcine liver was kept stationary for 2 h before microwave ablation. The starting temperature in our experiment was 22.97 °C, which was close to room temperature. Figure 5a shows that the fiber optic temperature probe was inserted parallelly to microwave ablation antenna. The horizontal distance between the probe and the antenna tip was 6 mm. Figure 5b shows the phase difference image calculated by k space data before and after microwave ablation. The microwave region and temperature difference values could be observed from the quantitative MR temperature mappings during microwave ablation procedure (Fig. 5d–i). With the increasing of ablation time, the area and temperature difference values of ablation region were increasing.

**Fig. 6:**
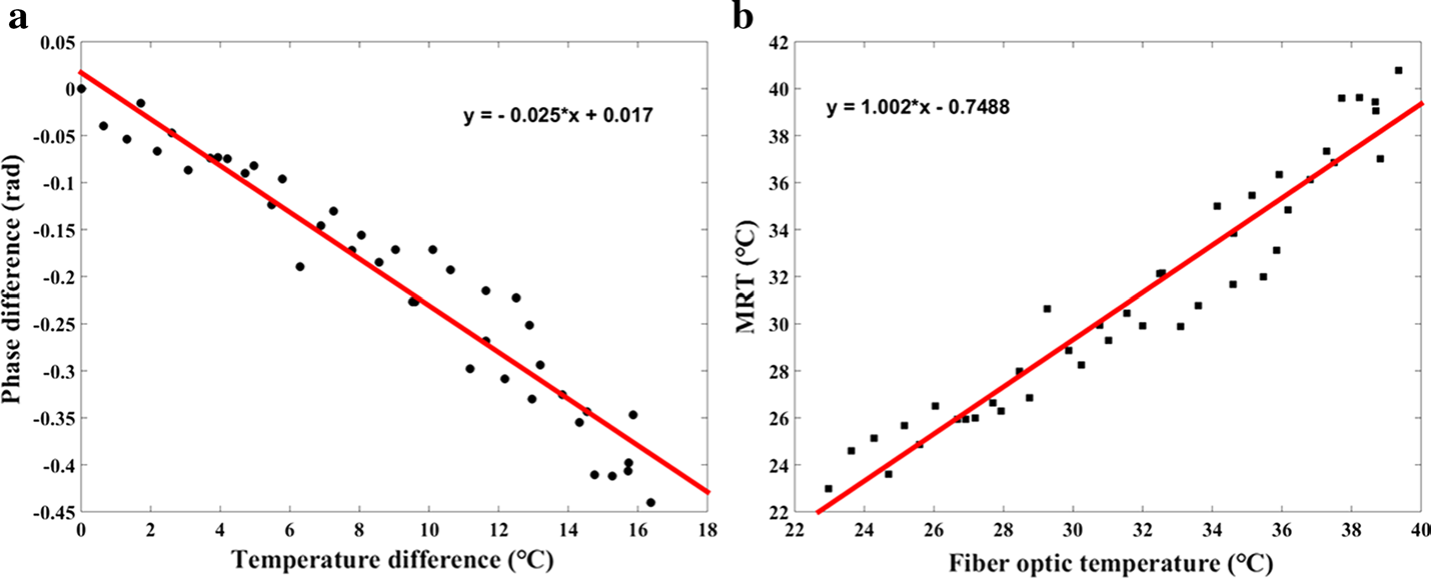
Phase and temperature changes of the ex vivo porcine liver MW ablation experiments. **a** Phase difference was a function of temperature difference. **b** Actual temperature values recorded by fiber optic temperature sensor were plotted versus temperature values measured with the phase-drift correction PRFS with first-order polynomial fitting method

**Fig. 7:**
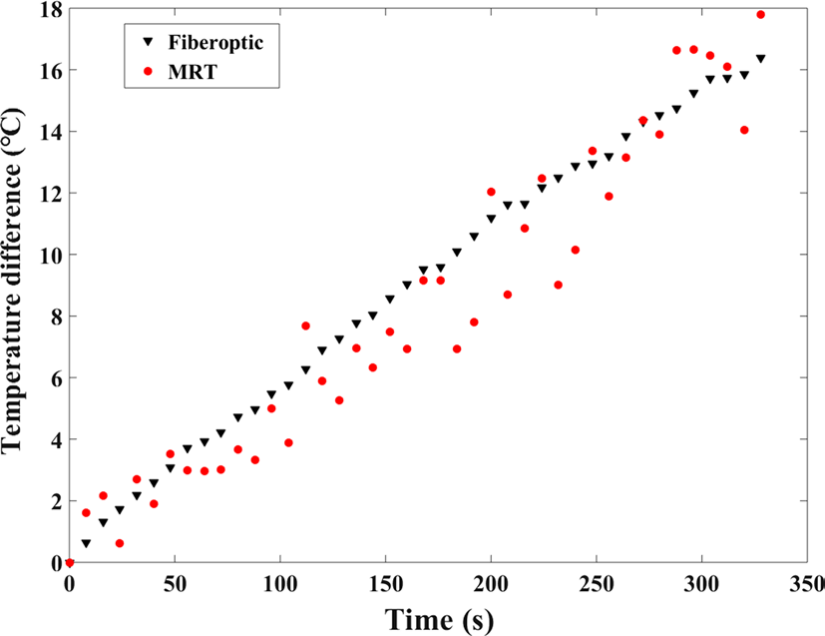
Relative temperature difference measured by phase-drift correction PRFS with first-order polynomial fitting method (red circle) versus that recorded by fiber optic temperature sensor (black triangle) at the same position for the ex vivo porcine liver MW experiments

Phase difference was plotted versus temperature difference recorded by fiber optic probe in Fig. 6a. The slope of the linear fit was − 0.0025. Thus, the calculation of PRF thermal coefficient yielded − 0.0088 ppm/°C for ex vivo porcine liver, which was applied for the following MW temperature difference measurements. Figure 6b shows actual temperature recorded by fiber optic temperature sensor versus temperature measured with the phase-drift correction PRFS with first-order polynomial fitting method. There was a good correlation between temperature measured with MRT and temperature recorded by fiber optic temperature sensor (ρ = 0.9737; P = 0, Spearman test). The slope of the linear fit was 1.002. Figure 7 represented relative temperature difference measured by phase-drift correction PRFS with first-order polynomial fitting method (red circle) versus that recorded by fiber optic temperature sensor (black triangle) at the same position. During microwave ablation procedure, 42 temperature points were recorded and the relative temperature difference values of MRT and fiber optic probe changed from 0 to 17 °C. The temperature errors between fiber optic probe and MRT of all but six temperature points were less than 2 °C. On average, the RMSE of all temperature points was 1.49 °C.

### In vivo human brain experimental results

**Fig. 8:**
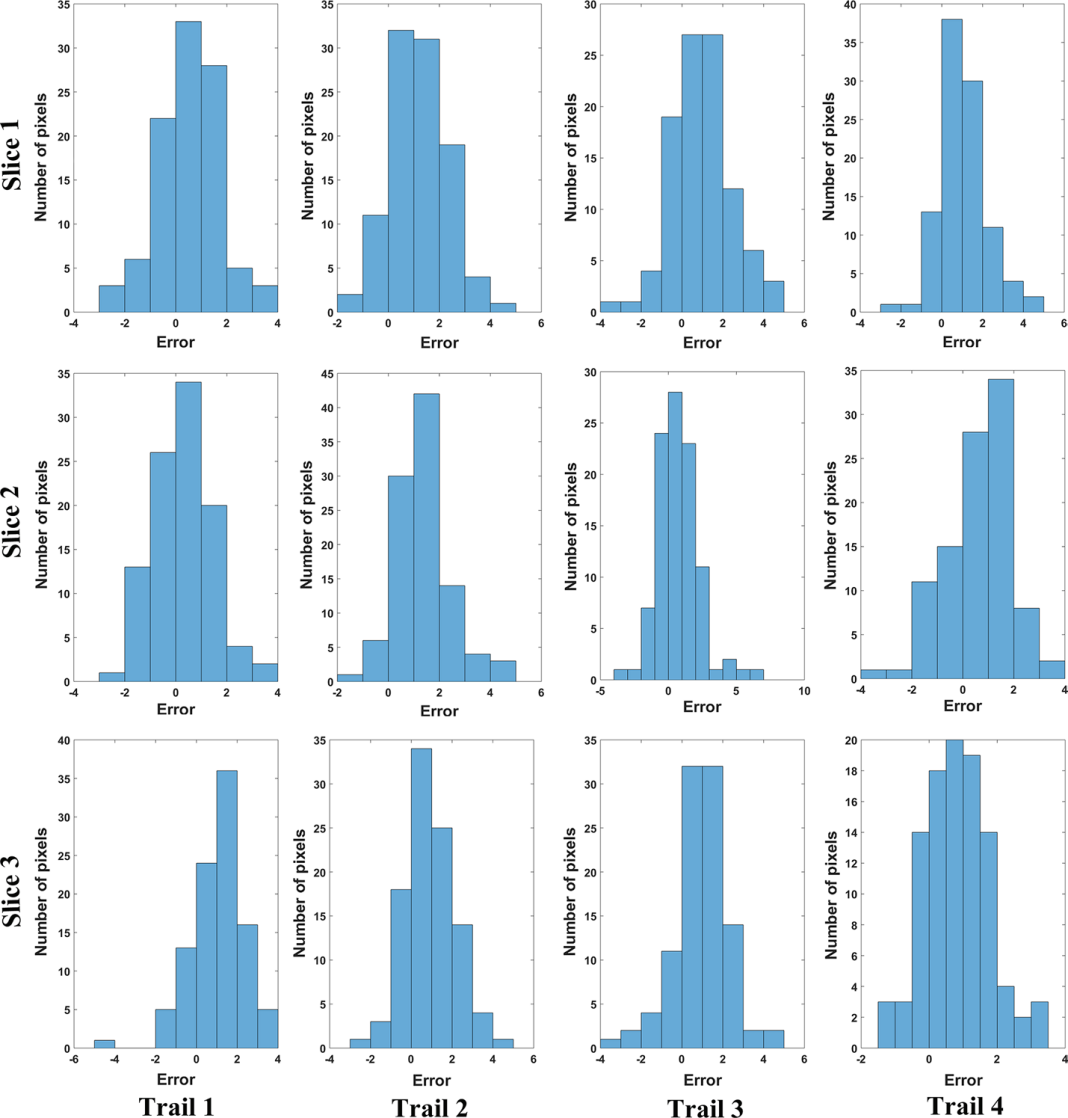
Temperature error distribution histogram of each pixel within the inner ROI (100 pixels) for body temperature brain experiments (Volunteer #1). Note that all units of error were °C

The mean and SD value of the temperature RMSE for the three volunteer brain experiments were listed in Table 1. Without phase-drift correction, the mean value of temperature uncertainty computed by conventional PRFS were all larger than that calculated by phase-drift correction PRF thermometry. The mean and SD values the temperature RMSE of four trials in each slice calculated by first-order polynomial fitting were 1.51 ± 0.16, 1.49 ± 0.22 and 1.57 ± 0.15 °C for volunteer #1, 1.12 ± 0.10, 1.29 ± 0.28 and 0.95 ± 0.17 °C for volunteer #2, 1.34 ± 0.13, 1.79 ± 0.25 and 1.32 ± 0.24 °C for volunteer #3, respectively. Compared with the polynomial of second- to sixth-order, the temperature error calculated by the phase-drift correction PRFS with first-order polynomial fitting was the smallest. The algorithm of phase-drift correction PRFS thermometry was stable because the results showed that no significant difference in the temperature errors of each trial for three volunteers (All P values > 0.05). Figure 8 shows the temperature error distribution histogram for each pixel within the inner ROI (100 pixels) obtained by the phase-drift correction PRFS with first-order polynomial fitting method on volunteer #1.

**Table 1:** The mean and SD value of temperature errors for human brain experiments

Slice	Volunteer	PRFS	First	Second	Third	Fourth	Fifth	Sixth
1	#1	7.68 ± 0.56	1.51 ± 0.16	2.90 ± 0.21	2.91 ± 0.21	4.67 ± 0.31	4.70 ± 0.31	5.88 ± 0.41
	#2	6.40 ± 0.65	1.12 ± 0.10	2.35 ± 0.23	2.37 ± 0.22	3.79 ± 0.40	3.80 ± 0.40	4.86 ± 0.52
	#3	6.99 ± 0.91	1.34 ± 0.13	2.69 ± 0.28	2.72 ± 0.29	4.22 ± 0.47	4.23 ± 0.47	5.29 ± 0.67
2	#1	7.79 ± 0.54	1.49 ± 0.22	2.73 ± 0.30	2.74 ± 0.29	4.47 ± 0.34	4.47 ± 0.35	5.80 ± 0.40
	#2	6.50 ± 0.58	1.29 ± 0.28	2.27 ± 0.28	2.27 ± 0.28	3.64 ± 0.37	3.64 ± 0.37	4.76 ± 0.48
	#3	7.30 ± 1.02	1.79 ± 0.25	2.82 ± 0.37	2.84 ± 0.37	4.35 ± 0.65	4.35 ± 0.65	5.57 ± 0.81
3	#1	7.88 ± 0.32	1.57 ± 0.15	2.92 ± 0.15	2.92 ± 0.15	4.65 ± 0.17	4.65 ± 0.16	5.91 ± 0.23
	#2	6.23 ± 0.32	0.95 ± 0.17	1.91 ± 0.10	1.91 ± 0.10	3.29 ± 0.19	3.30 ± 0.19	4.47 ± 0.26
	#3	6.53 ± 0.63	1.32 ± 0.24	2.19 ± 0.36	2.19 ± 0.36	3.59 ± 0.46	3.63 ± 0.47	4.83 ± 0.56

All units of temperature errors were in °C

## Discussion

For clinical application, MRT should provide good temporal and spatial resolution to precisely monitor the temperature changes during thermal therapies. In this study, we have demonstrated the capability and stability of the phase-drift correction PRFS thermometry via unheated phantom and ex vivo porcine liver experiments on a low-field MR scanner. Ex vivo porcine liver MW experiments also validated that phase-drift correction PRFS with first-order polynomial fitting can be applied to guide hyperthermia procedures and monitor temperature changes at low-field MR scanner.

In our study, the mean temperature errors for phantom experiments were 1.54, 1.46 and 1.47 °C for each slice (Fig. 4a), which were better than the results (2.5, 3.3 °C) obtained on a 0.2 T interventional MRI scanner [32, 35]. Temperature uncertainty for ex vivo porcine liver experiments in this study (1.49 °C) maintained an accuracy similar to the results reported by Harth et al. on a 1.5 T MRI scanner (1.50 °C) [41]. Human brain studies showed that the temperature error obtained by the phase-drift correction PRFS thermometry was larger than that calculated by the phase gradient method, and smaller than that obtained by the phase finite difference model from a previous study [40]. The distinction of results could be acceptable and reasonable due to the obvious difference in field strengths.

Interventional open MR scanners typically operate at a low-field strength in the range of 0.2–1.0 T [42]. With wider bore, low-field MR scanners are relatively suitable for performing MR guided thermal therapy temperature monitoring, because they are open system with access to the patients. Another meritorious advantage is that low-field MR scanners are cheaper than these high-field MR systems [34, 38]. Compared with high-field MR systems, lower signal-to-noise (SNR), longer scanning time and less homogeneous main magnetic fields are the major disadvantages of low-field strength MR scanner [39]. Nevertheless, temperature sensitivity is positively correlated with echo time and main magnetic field strength. For the same temperature sensitivity, the echo time for 0.35 T should be about 8.6 times longer than that needed at 3.0 T. Longer acquisition time is required to obtain acceptable temperature sensitivity at low-field. However, high temporal resolution of MRT is also imperative for temperature surveillance during thermal therapies. With reference to the previous studies [10, 43], we selected TR/TE = 50/30 ms for this studies in order to obtain acceptable temporal resolution and accurate temperature measurements during microwave ablation at low-field strength. The temporal resolution of low-field MRT in this study (6.4 ms) was not as good as high-field MRT, whose temporal resolution was generally about 5 ms [11, 40, 44]. Low-energy, long-time thermal therapy could compensate for the drawback of the temporal resolution for low-field scanners. In addition, the temporal resolution of the proposed method was superior to MR temperature mapping based on T1 [33].

During unheated experiments, we found that first-order polynomial fitting achieved the smallest RMSE(Fig. 4 and Table 1). A study by Rieke et al. which showed a fourth-order polynomial could approximate the background phase in unheated phantom, was different from the results of this study [10]. The fact that the best order of polynomial fitting is dependent on the scanned object, the homogeneity of the magnetic field and the ROI selection may explain the difference between these two studies. The differences may be caused by the differences of homogeneity of the magnetic field between permanent magnet MR and superconducting MR. Several theoretical facts may also account for the differences of the optimal fitting polynomial order for phase-drift correction between low-field and high-field MR system. Firstly, the static magnetic field drift should be much less in superconducting magnet than permanent magnet [45]. Secondly, the vast majority of high-field MR scanners are of cylindrical design and have their main magnetic fields directed along the bore of the scanner, while the permanent MR scanners have their fields directed vertically or horizontally [46]. Thirdly, the main magnetic field of permanent MR scanners is less uniform than that of superconducting scanners [47]. Last but not least, signal-to-noise ratio (SNR) is approximately proportional to field strength even though a lot of complex factors may affect the image quality [48].

In this study, we set the size of FOV and acquisition matrix according to the different shape and size of the scanning object. Due to the different FOV and acquisition matrix, the size of the ROI could not be kept constant throughout all the experiments. In order to investigate the influence of ROI selection, we analyzed the temperature errors by selecting three different ROIs in the unheated porcine liver experiments. As the ratio between the numbers of inner and outer ROI pixels increased, the temperature RMSE in the inner ROI became larger (Fig. 4b). For comparison with high-field experimental results, the size of ROIs in human brain experiments was the same as that of Zou et al. [40].

T1 and PRF methods are commonly used for noninvasive MRT at low-field strength. Germain et al. accomplished in vivo temperature mapping using T1 and M_0_ method at a 0.23 T MR scanner [33]. In contrast, the feasibility of temperature measurement using PRF phase mapping had also been confirmed by means of a series of published literatures [10, 32]. In this study, we apply the PRFS to calculate temperature mapping on the basis of the fact that PRF tends to provide fast and accurate temperature changes during thermal ablation. Future studies will also focus on studying the accuracy and capability of T1 thermometry on our 0.35 T system.

The PRF thermal coefficient in our microwave ablation experiments was − 0.0088 ppm/°C for ex vivo porcine liver, which was different from the nominal value (Fig. 6a). Peters et al. demonstrated that there was no tissue type dependence of the PRF thermal coefficient via measurements on freshly excused animals tissues [22]. Nevertheless, a great range of PRF thermal coefficient had been reported in several literatures, e.g., − 0.0135 ppm/°C for a porcine liver, − 0.0146 ppm/°C for rat muscle, − 0.0088 ppm/°C for rabbit brain and − 0.0067 ppm/°C for canine muscle, etc. [24, 49–51]. Several mechanisms, including geometry of the object, volume magnetic susceptibility and electromagnetic properties, may account for the discrepancies in PRF thermal coefficient [22, 52].

The presented study has several limitations. Firstly, only in vitro experiments were involved in this study due to lack of clinical experience. More in vivo animal and volunteer studies are required to validate the feasibility and stability of the phase-drift correction PRFS thermometry during hyperthermia procedures. Moreover, the proposed method is not suitable for monitoring the temperature changes of all tissues, such as adipose tissue.

## Conclusions

In this preliminary study, in vivo and ex vivo experiments have demonstrated that the phase-drift correction PRFS thermometry with first-order polynomial fitting method could be one reliable and practical technique to monitor the temperature changes during microwave ablation procedure in a 0.35 T open-configuration whole-body MR scanner. With stable temperature accuracy and acceptable temporal resolution, low-field MR guided temperature mapping may become a reliable and competitive tool for monitoring thermal therapy procedures.
